# Characterisation of Four New Genes in the Ovine KAP19 Family

**DOI:** 10.3390/ijms26146863

**Published:** 2025-07-17

**Authors:** Lingrong Bai, Huitong Zhou, Jianning He, Jinzhong Tao, Guo Yang, Jon G. H. Hickford

**Affiliations:** 1 International Wool Research Institute, Faculty of Animal Science and Technology, Gansu Agricultural University, Lanzhou 730070, China; lingrong.bai@lincolnuni.ac.nz (L.B.); huitong.zhou@lincoln.ac.nz (H.Z.); tao_jz@nxu.edu.cn (J.T.); 2 Gene-Marker Laboratory, Faculty of Agriculture and Life Sciences, Lincoln University, Lincoln 7647, New Zealand; 3 Yellow River Estuary Tan Sheep Institute of Industrial Technology, Dongying 257400, China; yangguo@lzb.ac.cn; 4 College of Animal Science and Technology, Qingdao Agricultural University, Qingdao 266109, China; hexingxing104@163.com; 5 College of Animal Science and Technology, Ningxia University, Yinchuan 750021, China; 6 State Key Laboratory of Ecological Safety and Sustainable Development in Arid Lands, Northwest Institute of Eco-Environment and Resources, Chinese Academy of Sciences, Lanzhou 730000, China

**Keywords:** keratin-associated protein, *KRTAP19-1*, *KRTAP19-2*, *KRTAP19-4*, *KRTAP19-6*, variation, Yanchi Tan sheep

## Abstract

This study identified four new keratin-associated protein genes (*KRTAP19-n*) in sheep: *sKRTAP19-1*, *sKRTAP19-2*, *sKRTAP19-4*, and *sKRTAP19-6*. These genes are closely related to the previously identified sheep genes *KRTAP19-3* and *KRTAP19-5*, as well as to human *KRTAP19-n* genes. However, no clear orthologous relationships were found, suggesting complex evolutionary dynamics for this gene family. Extensive nucleotide sequence variation was observed across the four genes. *sKRTAP19*-1 had four variants, defined by four synonymous single-nucleotide polymorphisms (SNPs) and a variable number of “GGCTAC” hexanucleotide repeats. *sKRTAP19-2* had five variants involving seven SNPs, three of which were non-synonymous. *sKRTAP19-4* had five variants with nine SNPs (three being non-synonymous) and a three-nucleotide deletion. *sKRTAP19-6* had eight variants, defined by 13 SNPs and a two-nucleotide consecutive substitution, with four of the SNPs being non-synonymous. One distinct variant each of *sKRTAP19-4* and *sKRTAP19-6* was found exclusively in Yanchi Tan sheep, with seven unique nucleotide differences compared to other variants. These unique variants were identical to the Romanov sheep genome in the region amplified (excluding the primer binding regions), suggesting a shared ancestral origin. The findings highlight considerable genetic diversity in ovine *KRTAP19-n* and lay a foundation for future research into their role in regulating wool fibre characteristics.

## 1. Introduction

Wool, with its unique properties, is experiencing a resurgence as a sustainable natural fibre because its recyclability and renewable nature make it an environmentally friendly choice in an era increasingly focused on sustainability [1,2]. However, wool faces challenges due to the variability of the fibre, which can limit its utility and value [3]. Addressing this issue requires ongoing effort to improve and refine wool traits through selective breeding approaches.

At the molecular level, wool is primarily composed of wool keratins and keratin-associated proteins (KAPs) [4]. The wool keratin genes are reported to be fully catalogued, with 10 type I keratin genes and seven type II keratin genes [5], while the cataloguing of KAP genes (referred to as *KRTAPs*) in sheep is nearing completion, with 102 *KRTAPs* reported in the genome [6]. While all the wool keratin genes appear to have been identified, only a fraction of *KRTAPs* have been characterised [7].

Research on ovine *KRTAPs* has primarily focused on the high-glycine/tyrosine (HGT)-KAP group, driven by their variability in abundance both within and across species [8], as well as their observed reduction in expression in the Merino felting lustre mutant [9]. The HGT-*KRTAPs* are also among the earliest expressed genes in the developing wool fibre [5,10]. This suggests they regulate wool fibre properties, with studies revealing associations between variation in individual HGT-*KRTAPs* and variation in wool traits [6]. The annotation of the sheep keratin and KAP genes, along with their patterns of expression [5], has revealed some of the complexity of KAP and keratin expression in the wool follicle, but studies of this kind are hampered by the absence of knowledge of how many KAP genes exist. This is, in part, why a study of the kind we describe below is necessary. Equally, further analysis of phenotypic correlations is required if we are to create a better understanding of their role in regulating wool fibre characteristics.

The HGT-KAP group in humans comprises seven families: KAP6-KAP8, KAP19-KAP22 [11]. While gene members from all these HGT-KAP families have been investigated in sheep [6], the KAP19 family remains largely unexplored. It appears to be the largest family in the HGT-KAP group, with seven members identified in humans [12]. In sheep, only two genes from this family, *KRTAP19-3* [13] and *KRTAP19-5* [14], have been characterised to date, and this suggests that there may be additional *KRTAP19-n* genes that are yet to be identified and studied.

In this study, we aim to identify and characterise additional members of the ovine KAP19 family. We will investigate sequence variation in these newly identified KAP19 gene members and assess their potential association with wool traits in Chinese Tan sheep, a breed known for its distinctive wool properties, including having a “spring-like” crimp that is observed in the early stages of growth.

## 2. Results

### 2.1. Identification of New KRTAP19-n on the Sheep Genome

A BLAST search using the ovine *KRTAP19-3* coding sequence (GenBank accession number PV457914) to search the sheep genome assembly (ARS-UI_Ramb_v3.0 (GCF_016772045.2)) chromosome 1 sequence (RefSeq NC_056054.1), revealed six matches. Among these matches, two sequences corresponded to the previously identified genes *KRTAP19-3* and *KRTAP19-5*, while the remaining four were novel. These matches contained ORFs, that were localised as follows: ORF1 (g.125973979_125974197, 219 nucleotides, putatively 72 amino acids, identity = 86%, E-value = 8 × 10^−59^), ORF2 (g.125978223_125978444, 222 nucleotides, putatively 73 amino acids, identity = 86%, E-value = 1 × 10^−61^), ORF3 (g.125994648_125994869, 222 nucleotides, putatively 73 amino acids, identity = 87%, E-value = 6 × 10^−65^) and ORF4 (g.126001763_126001984, 222 nucleotides, putatively 73 amino acids, identity = 87%, E-value = 6 × 10^−65^).

**Figure 1:**
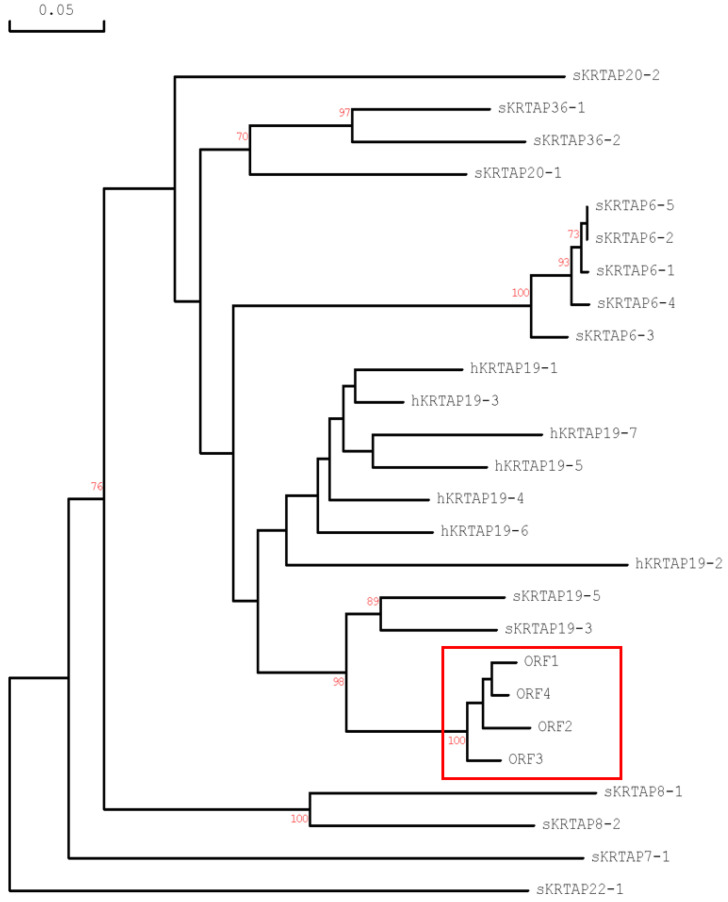
Phylogenetic tree of the four new open reading frames (ORFs) identified in this study, and other ovine HGT-*KRTAPs* and human *KRTAP19-n* sequences. The new ORFs are highlighted in boxes. The sheep *KRTAPs* are annotated with the prefix “s”, while the human genes are identified by the prefix “h”. Bootstrap confidence values (shown in red) are displayed at the forks, with only values over 70% shown. The scale bar represents a rate of 0.05 nucleotide substitutions per site.

Phylogenetic analysis of these four ORFs suggested they formed a distinct cluster that was closely related to ovine *KRTAP19-3* and *KRTAP19-5* (Figure 1). They also shared similarity with a cluster encompassing all the human *KRTAP19-n* genes and not other sheep HGT-*KRTAPs*. Accordingly, the ORFs were thought to represent new members of the KAP19 family in sheep.

**Figure 2:**
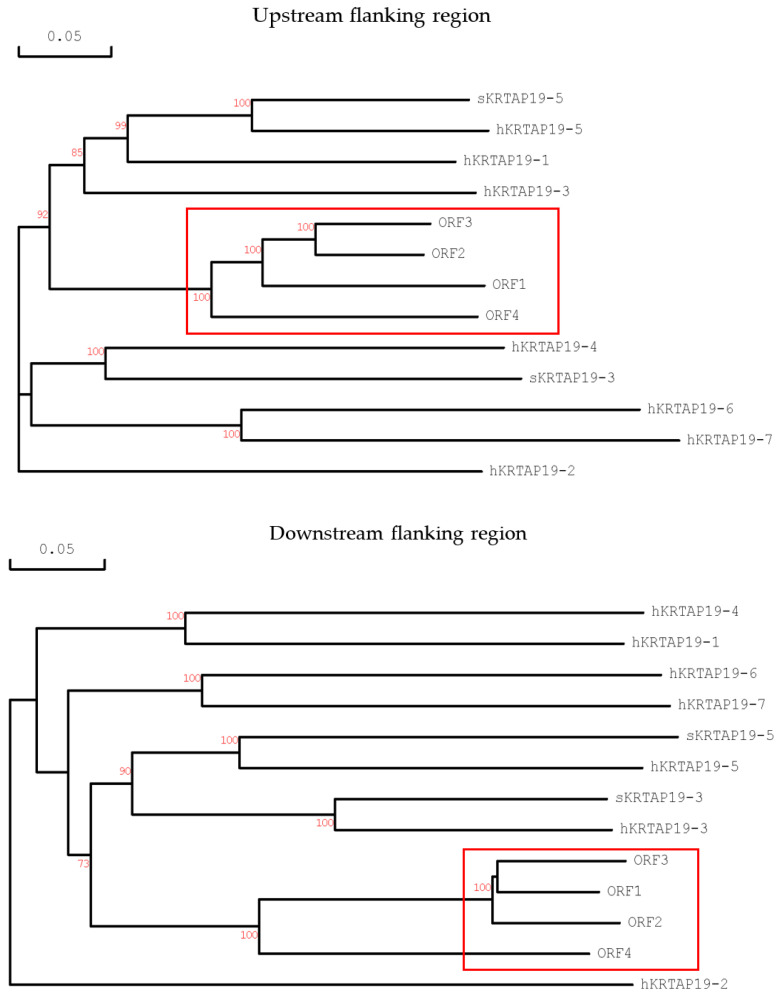
Phylogenetic analysis of the upstream and downstream flanking regions of the four newly identified open reading frames (ORFs), in comparison to other *KRTAP19-n* from sheep and humans. The new ORFs are highlighted within a box. Sheep genes are annotated with the prefix “s”, while human genes are identified by the prefix “h”. Bootstrap confidence values (shown in red) are displayed at the forks, with only values over 70% shown. The scale bars represent a rate of 0.05 nucleotide substitutions per site.

To further test these findings, phylogenetic analyses were undertaken of the 1 kb upstream and downstream flanking regions of the ORFs. However, these did not reveal anything more about the relationships with individual *KRTAP19-n* genes from humans (Figure 2), making it difficult to determine their corresponding human orthologs. As a consequence, these ORFs were named *sKRTAP19-1*, *sKRTAP19-2*, *sKRTAP19-4*, and *sKRTAP19-6*, given that *sKRTAP19-3* and *sKRTAP19-5* have already been identified. It is important to note that *sKRTAP19-1* may not necessarily correspond directly to human *KRTAP19-1*, and likewise for *sKRTAP19-2*, *sKRTAP19-4*, and *sKRTAP19-6*.

### 2.2. Sequence Variants and Polymorphisms in sKRTAP19-n

**Figure 3:**
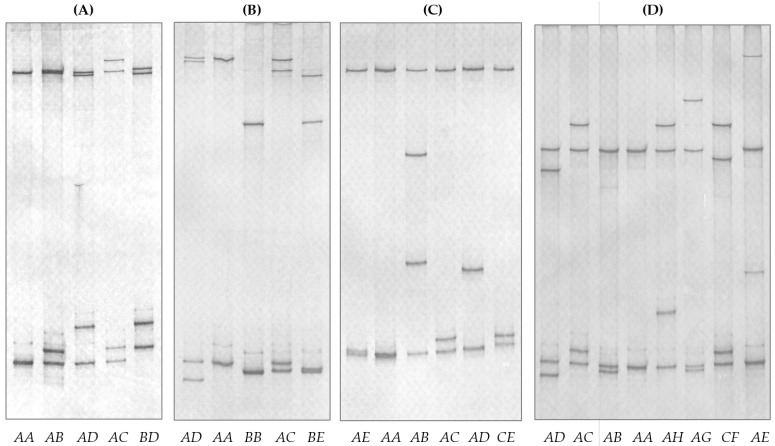
PCR-SSCP patterns of four ovine *KRTAP19-n* genes. (**A**) Four different variants (*A* to *D*) are observed for *sKRTAP19-1*, (**B**) five different variants (*A* to *E*) for *sKRTAP19-2*, (**C**) five different variants (*A* to *E*) for *sKRTAP19-4*, and (**D**) eight different variants (*A* to *H*) for *sKRTAP19-6*. These variants appear in both homozygous and heterozygous sheep, with the genotypes identified shown below the gel patterns.

**Figure 4:**
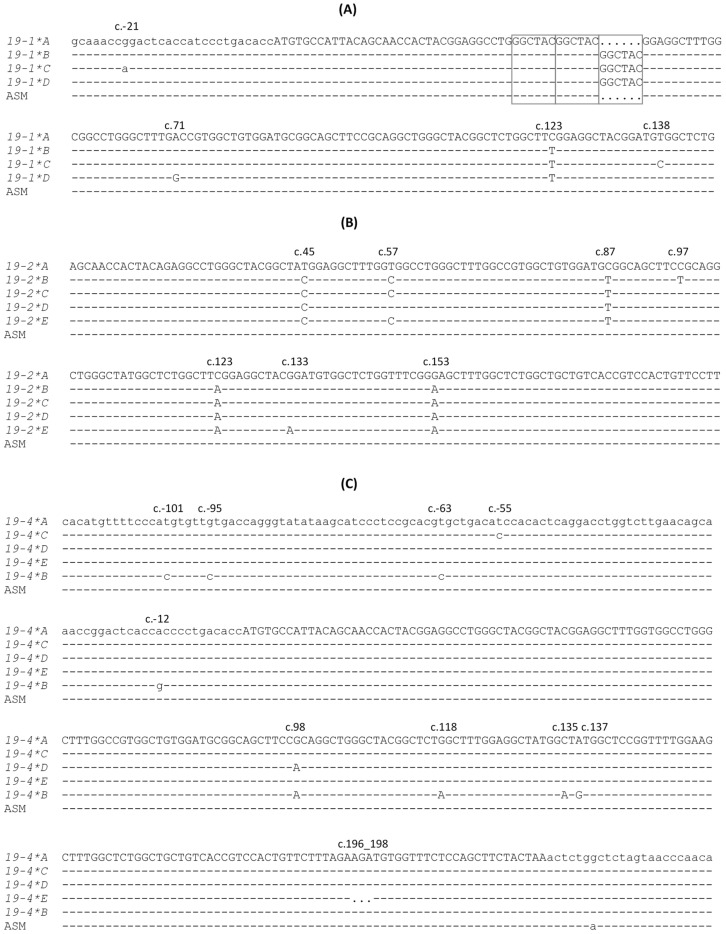

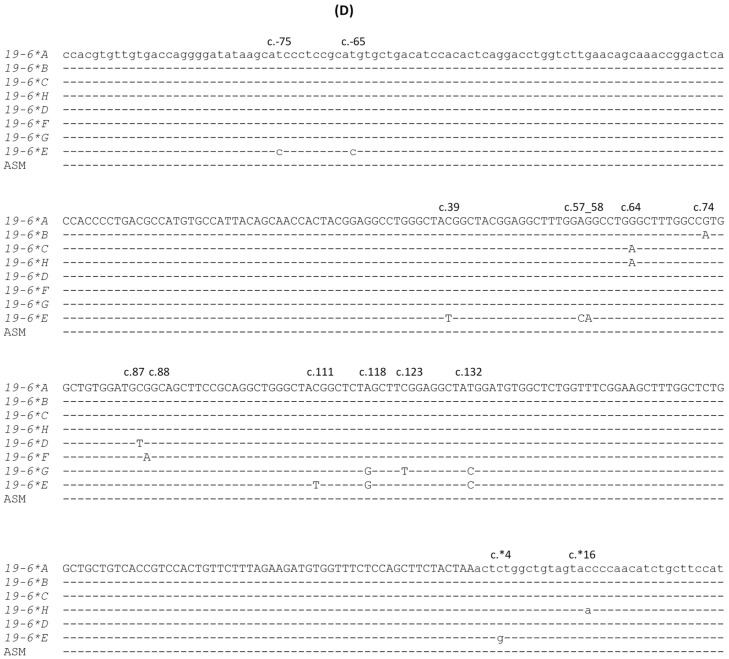
Nucleotide sequence alignments of the variant sequences along with the genome assembly sequence for the four ovine KAP19 genes. The KAP19 genes shown are (**A**) *KRTAP19-1*, (**B**) *KRTAP19-2*, (**C**) *KRTAP19-4*, and (**D**) *KRTAP19-6*. The variant names are presented in a shortened form (i.e., KRTAP19-1*A is represented by 19-1*A), and the genome assembly sequence for each gene is labelled as ASM. Nucleotides within the coding region are in uppercase letters, while those outside the coding regions are in lowercase. Nucleotides identical to the top nucleotide sequence are indicated with dashes, while dots represent inferred gaps where nucleotides may be absent or deleted. The boxed sequences represent repeats that have variation in repeat number. The positions of all the sequence variations are shown above the sequences.

PCR single-strand conformation polymorphism (PCR-SSCP) analysis identified four banding patterns for *sKRTAP19-1* (Figure 3A), corresponding to four sequence variants (Figure 4A). These variant sequences exhibited four single-nucleotide polymorphisms (SNPs) and a length variation. One SNP was located 21 bp upstream of the start codon, while the remaining three were located in the coding region and were synonymous. The length variation resulted from differences in the copy number of a “GGCTAC” element, with variant *A* containing two copies and the others having three. Variant *A* is identical to the chromosome 1 genome assembly sequence (NC_056054.1).

For *sKRTAP19-2*, five PCR-SSCP patterns were detected (Figure 3B), leading to the identification of six sequence variants (Figure 4B). Seven SNPs were found, all within the coding region, including three non-synonymous substitutes: c.97C/T (p.Arg33Sys), c.123C/A (p.Phe41Leu), and c.133G/A (p.Gly45Arg). Variant *A* matches the sheep genome assembly sequence (NC_056054.1).

Five PCR-SSCP patterns were revealed for *sKRTAP19-4* (Figure 3C), corresponding to five sequence variants (Figure 4C). Nine SNPs and a three-nucleotide deletion (AGA) were identified. Four SNPs were within the coding region, and three were non-synonymous: c.98G/A (p.Arg33His), c.118G/A (p.Gly40Ser), and c.137A/G (p.Tyr46Cys). The deletion c.196_198del preserved the reading frame, but would putatively result in the loss of an arginine residue. None of the identified *sKRTAP19-4* variant sequences are identical to the sheep genome assembly (NC_056054.1), but variant *A* is the closest match, differing by a single nucleotide downstream of the coding region.

For *sKRTAP19-6*, eight PCR-SSCP patterns were detected (Figure 3D), revealing eight sequence variants (Figure 4D). A total of 13 SNPs and a 2-nucleotide consecutive substitution (described as a deletion-insertion according to HGVS recommendations) were found. Two SNPs were located upstream and two downstream of the coding region, while the remaining nine were within the coding region. Among these, four were non-synonymous: c.64G/A (p.Gly22Ser), c.74G/A (p.Arg25His), c.88G/A (p.Gly30Ser), and c.118A/G (p.Ser40Gly). The deletion-insertion c.57_58delinsCA would putatively lead to the amino acid change p.Gly20Ser. Variant *A* is identical to the sheep genome assembly (NC_056054.1).

The frequencies of the identified *sKRTAP19-1*, *sKRTAP19-2*, *sKRTAP19-4*, and *sKRTAP19-6* variants in the sheep analysed in this study are summarised in Table 1. The heterozygosity and polymorphism information content (PIC) values for each gene are also provided in the table. The gene *sKRTAP19-4* exhibited a low level (<25%) of heterozygosity and PIC, while *sKRTAP19-1* had a medium level (25–50%), and *sKRTAP19-2* and *sKRTAP19-6* had high levels (>50%) of heterozygosity and PIC.

The sequences of all these variants have been deposited in GenBank under the following accession numbers: PV457920-PV457923 (*sKRTAP19-1* variants *A* to *D*), PV457924-PV457928 (*sKRTAP 19-2* variants *A* to *E*), PV457929-PV457933 (*sKRTAP19-4* variants *A* to *E*), and PV457934-PV457941 (*sKRTAP19-6* variants *A* to *H*).

### 2.3. Characterisation of Distinctive sKRTAP19-n Variants and Their Sequence Comparison

Among the *sKRTAP19-n* sequences identified, two unusual sequence variants were observed—one in *sKRTAP19-4* and the other in *sKRTAP19-6*. Variant *B* of *sKRTAP19-4* contains seven unique nucleotide sequences compared to other variants of the same gene, while variant *E* of *sKRTAP19-6* also contains seven unique nucleotide sequences (Figure 4). Both variants were present in heterozygous forms and were exclusively found in 18 Yanchi Tan sheep derived from six sires. These variants were consistently co-inherited in these Yanchi Tan sheep (i.e., all sheep carrying sKRTAP19-4*B also carried sKRTAP19-6*E, or vice versa), suggesting a common ancestral origin.

A BLAST search against the sheep chromosome 1 genome assembly sequence (NC_056054.1) revealed that these variants exhibit high sequence similarities to *sKRTAP19-4* and *sKRTAP19-6*, respectively, and subsequent BLAST searches against known sheep whole-genome shotgun contigs revealed that the sequences of both variants match the Romanov sheep genome of chromosome 1 (Sequence ID: JAMFTK010000001.1). Variant sKRTAP19-4*B was identical to positions 130794501-130794916 of this genome sequence, while sKRTAP19-6*E matched to positions 130801575-130802036 with the exception of two nucleotide differences in the primer binding regions: one at the 9th nucleotide from the 5′ end of the forward primer, and the other one at 8th nucleotide from the 5′ end of the reverse primer.

## 3. Discussion

In this study, we identified four novel *KRTAP19-n* genes in sheep, expanding the known members of the ovine *KRTAP19* gene family to six. Phylogenetic analysis revealed that the newly identified genes form a distinct cluster, which is closely related to ovine *KRTAP19-3* and *KRTAP19-5*, more broadly related to the human *KRTAP19-n* genes, but separate from other known ovine HGT-*KRTAPs*. This suggests that the newly identified genes represent members of the ovine *KRTAP19* family and that the ovine *KRTAP19* gene family likely shares a common evolutionary origin with the human *KRTAP19* gene cluster.

When genes from a family exhibit higher sequence similarity within a species than between species, it is called concerted evolution. In sheep *KRTAP* genes, this effect has been described for the KAP1 [15], KAP6 [16], and KAP13 [17] families, as well as for *KRTAP19-3* [13] and *KRTAP19-5* [14]. This pattern of evolution is typically observed within the coding region for *KRTAP* genes, while divergent evolution is often seen in the flanking regions. These divergent patterns enable the matching of sheep orthologs to the human *KRTAP* genes [14], but for the four newly identified *KRTAP19-n* genes, concerted evolution appears to extend into the flanking regions. Analysis of these regions did not reveal obvious orthologous relationships with specific human *KRTAP19* genes, and this suggests that these *sKRTAP19-n* genes might have evolved in a different way from other ovine KAP families, as well as from *KRTAP19-3* and *KRTAP19-5* within the KAP19 family. The KAP19 family may, therefore, have come about by a distinct evolutionary path, with this potentially having functional implications for wool/hair fibre formation and adaptation.

Extensive sequence variation was observed among the newly identified *sKRTAP19-n* genes, with multiple nucleotide substitutions and length variations. Some nucleotide substitutions resulted in amino acid sequence changes, while length variations, such as the “GGCTAC” repeat polymorphism in *sKRTAP19-1*, and c.196_198del in *sKRTAP19-4*, may result in altered protein length. These changes could influence protein structure, function, the assembly of the wool fibre, and, accordingly, possibly wool fibre characteristics. Variations in the upstream and downstream regions of the coding region may also affect gene expression [18], while synonymous nucleotide substitutions can affect mRNA stability, translation, and co-translational protein folding [19,20], with these all potentially exerting functional effects on wool fibres.

Despite the genes being clustered on the same chromosome, the extent and nature of the variation differed among these genes. This is consistent with findings for other clustered *KRTAP* genes [7,17], and it suggests that individual genes may have different functional roles and therefore be subject to different evolutionary forces. The detection of length variation in *sKRTAP19-1* and *sKRTAP19-4* as a consequence of repeat sequence variation is also consistent with what is observed for other *KRTAP* genes in sheep [21] and goats [22].

With the exception of *sKRTAP19-4*, a variant identical to the sheep genome assembly sequence was identified for the other three *sKRTAP19* genes in the sheep studied. None of the *sKRTAP19-4* variants matched the genome assembly sequence, which would suggest the potential for additional sequence variation to be found, should more sheep be studied, albeit it could also possibly reflect inaccuracies in the reference genome sequences rather than the novel sequences discovered here. Future investigation should clarify whether this discrepancy is due to sequencing or assembly error, or whether it reflects genuine genetic variation, the latter suggesting that more sheep of differing breeds need to be typed.

A particularly interesting finding was the identification of distinct sequence variants within *sKRTAP19-4* and *sKRTAP19-6*, which were characterised by a high number of unique nucleotides distributed across the two genes. Variant *B* of *sKRTAP19-4* and variant *E* of *sKRTAP19-6* ecah contained seven unique nucleotide sequences within relatively short DNA fragments (<500 bp), a level of sequence variation not previously described in other *KRTAPs* from sheep and goats [7,23,24,25]. The amplification of sKRTAP19-6*E was slightly weaker than that of the other variants, raising the possibility that these sequences resulted from non-specific amplification of other *KRTAPs*, but if non-specific amplification was responsible, one would expect to observe more than two variants in heterozygous sheep. This was not the case, as no heterozygous individuals carrying these variants displayed more than two sequences (typically four bands) on the SSCP gels. As these variants were only found in heterozygous forms, if they had originated from other species, the second variant would also be expected to exhibit a different banding pattern compared to the sheep variants, but no such differences were observed. This suggests the variants are not the result of non-specific amplification or contaminating sequences from other species. BLAST searches identified matching sequences on the Romanov whole-genome shotgun contigs at loci corresponding to the positions of *KRTAP19-4* and *KRTAP19-6*, which strongly suggests that these sequences are genuine sequence variants of these genes. The presence of two nucleotide mismatches in the upstream and downstream primer binding region would explain the weaker amplification of sKRTAP19-6*E.

The identification of these two distinct variants in Yanchi Tan sheep, a population regarded as purebred and used for both breeding and breed conservation in China, suggests that genetics from distant breeds, such as the Romanov, may have been introgressed into the Yanchi Tan sheep population in the past through previously undocumented means, or vice versa. The Romanov breed is renowned for its high fertility and adaptability to colder temperatures, which may have historically led to it being crossed into Tan sheep, specifically to enhance these traits. Romanov sheep also exhibit unique fleece properties, including a favourable wool-to-hair ratio (4–10:1 in fibre numbers) and having wool fibres with a high scale frequency (14 scales/100 μm), making them particularly suitable for felt production [26,27]. Although originating in Russia, whole-genome analyses have shown that the Romanov sheep are genetically distinct from other Russian sheep breeds, with unique genetic sequences having been identified [28,29,30].

The discovery of distinct *sKRTAP19-4* and *sKRTAP19-6* variants in Romanov sheep is unsurprising, given their genetic distinctiveness, but no distinct variants have been identified in other *KRTAP* genes, including other KAP19 family members described here. This suggests that these variants of the two genes could potentially serve as unique genetic markers for Romanov and/or Yanchi Tan sheep.

While *sKRTAP19-4* and *KRTAP19-6* have distinct variants in the Yanchi Tan sheep studied, the other *KRTAP19* genes did not have breed-specific variants. This raises the question of why only *sKRTAP19-4* and *sKRTAP19-6* genes have such distinct variants? Are they associated with unique fibre or fleece properties, do they reflect selection for other unknown functions, or are they simply the result of insufficient sampling of sheep to find the unique variants elsewhere? Future studies on *KRTAP* gene variation, expression, and their effects on wool traits in Romanov sheep, Yanchi Tan sheep, and other breeds globally could provide further insight into the functional roles and evolution of the *KRTAP* genes.

Given the genetic difference in *KRTAP19* genes between sheep and humans, as well as the extensive variation observed among sheep, further research is needed to ascertain if these genes are expressed in wool follicles and where in wool fibre development. Research also needs to explore the functional consequences of the sequence variations.

## 4. Materials and Methods

### 4.1. Selection of Sheep and DNA Purification

A total of 305 sheep blood samples on TFN paper (Munktell Filter AB, Falun, Sweden) were collected from 32 different farms across New Zealand and China. These included Merino (*n* = 13), Corriedale (*n* = 11), New Zealand Romney (*n* = 9), Texel (*n* = 6), South Suffolk (*n* = 5), Poll Dorset (*n* = 3), and Coopworth (*n* = 3), as well as Chinese Tan sheep (*n* = 255) from Yanchi, China. All sheep from New Zealand were known to be stud sheep. This selection aimed to maximise the likelihood of capturing a wide range of sheep genetic material. All samples had been sent to a commercial DNA typing laboratory for routine gene testing; hence, ethics approval was not required for the blood collection. Purified DNA for PCR amplification was prepared from 1.2 mm punches of dried blood on the TFN paper using the procedure outlined by Zhou et al. [31].

### 4.2. BLAST Search of Sheep Genome Assembly

In a previous study [14], the coding sequence of ovine *KRTAP19-5* (Ensembl ENSOARG00020032146) was used in a BLAST search of a Rambouillet sheep genome assembly (ARS-UI_Ramb_v2.0; GCF_016772045.1), resulting in the identification of a sequence that was named *sKRTAP19-3*. Adapting this approach for this study, we used the coding sequence of ovine *KRTAP19-3* (GenBank accession number PV457914) to conduct a BLAST search of the sheep genome assembly ARS-UI_Ramb_v3.0 (GCF_016772045.2). Open reading frames (ORFs) that had similarity to the ovine *KRTAP19-3* sequence were considered to potentially be other members of the KAP19 family.

### 4.3. PCR Amplification and SSCP Analysis

Primers for amplifying the putative *KRTAP19-n* genes were designed based on the sequences flanking the open reading frames identified above. The sequences of these PCR primers are shown in Table 2, and they were synthesised by Integrated DNA Technologies (Coralville, IA, USA).

The PCR amplification was carried out in a 15 μL reaction containing the purified genomic DNA on the TFN paper punches, 150 μM of each dNTP (Bioline, London, UK), 0.25 μM of each primer, 2.5 mM Mg^2+^, 0.5 U of Taq DNA polymerase (Qiagen, Hilden, Germany), and 1× the reaction buffer provided with the enzyme. Thermal cycling conditions included an initial denaturation at 94 °C for 2 min, followed by 35 cycles of denaturation at 94 °C for 30 s, annealing at the temperatures specified in Table 2 for 30 s, extension at 72 °C for 30 s, and a final extension at 72 °C for 5 min. The thermal cycling was carried out in S1000 thermal cyclers (Bio-Rad, Hercules, CA, USA).

The PCR products were subjected to SSCP analysis. In brief, 0.7 μL aliquots of each amplicon were mixed with 7 μL of gel loading dye (0.025% bromophenol blue, 0.025% xylene-cyanol, 98% formamide, 10 mM EDTA). After denaturation at 95 °C for 5 min, the samples were rapidly cooled on wet ice and then loaded onto 14% acrylamide: bisacrylamide (37.5:1) gels (16 cm × 18 cm). Electrophoresis was carried out for 19 h using 0.5× TBE buffer in Protean II xi cells (Bio-Rad) under the conditions described in Table 2. Subsequently, the gels were silver-stained following the method described by Byun et al. [32].

### 4.4. DNA Sequencing and Sequence Analysis

PCR amplicons with different SSCP gel banding patterns from sheep that appeared to be homozygous for the target gene (typically, homozygous sheep produce two bands upon staining, corresponding to two single-strands of DNA), were sequenced in triplicate in both directions at the Lincoln University DNA sequencing facility (Lincoln University, Lincoln, New Zealand), using Sanger sequencing and the same primers as used for the PCR amplification. Variants that were only detected in heterozygous sheep were sequenced using a gel separation-based method described by Gong et al. [33]. Briefly, gel slices corresponding to the SSCP bands of the variants were excised, macerated, and the eluted single-strand DNA templates were used for re-amplification with the original primers. The resulting amplicons were then directly sequenced in triplicate using Sanger sequencing.

Sequence alignments, translation, and phylogenetic analysis were undertaken using DNAMAN XL (version 10, Lynnon BioSoft, San Ramon, California, CA, USA). Nucleotide sequences of all the identified ovine HGT-*KRTAPs* and human *KRTAP19-n* were obtained from GenBank, with accession numbers as follows: NM_001193399 (*sKRTAP6-1*), KT725832 (*sKRTAP6-2*), KT725837 (*sKRTAP6-3*), KT725840 (*sKRTAP6-4*), KT725845 (*sKRTAP6-5*), X05638 (*sKRTAP7-1*), X05639 (*sKRTAP8-1*), KF220646 (*sKRTAP8-2*), MH243552 (*sKRTAP20-1*), MH071391 (*sKRTAP20-2*), KX377616 (*sKRTAP22-1*), MK770620 (*sKRTAP36-1*), OR684903 (*sKRTAP36-2*), AJ457067 (*hKRTAP19-1*), NM_181608 (*hKRTAP19-2*), NM_181609 (*hKRTAP19-3*), NM_181610 (*hKRTAP19-4*), NM_181611 (*hKRTAP19-5*), NM_181612 (*hKRTAP19-6*), and NM_181614 (*hKRTAP19-7*).

### 4.5. Analysis of Genetic Diversity

Genetic diversity was assessed by calculating heterozygosity and PIC using an online calculator (https://www.genecalculators.net/pq-chwe-polypicker.html; accessed 4 March 2025). Variant frequencies were determined as the number of occurrences of each variant divided by the total number of variants observed in the sheep investigated and expressed as a percentage.

## 5. Conclusions

This study reports the identification of four additional *KRTAP19-n* genes in sheep. Although phylogenetically related to human *KRTAP19-n* genes, the sheep *KRTAP19-n* genes do not have exact orthologues in the human genome. This study also revealed considerable genetic variation within these genes, including nucleotide substitutions and length variations, with the nature and extent of variation differing among the genes. These findings enhance our understanding of the ovine *KRTAP19* genes and lay a foundation for future research into their role in regulating wool fibre characteristics.

## Figures and Tables

**Table 1:** Variant frequency and genetic diversity information of four ovine *KRTAP19-n* genes.

Gene ^1^	Variant Frequency (%)								Het ^2^ (%)	PIC ^3^ (%)
	*A*	*B*	*C*	*D*	*E*	*F*	*G*	*H*		
*19-1*	76.1	15.2	8.4	0.3					39.1	35.6
*19-2*	50.5	26.1	12.8	6.7	3.9				65.5	60.1
*19-4*	94.7	3.0	1.0	1.0	0.3				10.2	10.1
*19-6*	62.4	22.3	7.7	3.9	3.0	0.3	0.2	0.2	55.3	50.6

^1^ Gene names are shown in a shortened form (i.e., *KRTAP19-1* is represented as *19-1*). ^2^ Het: heterozygosity. ^3^ PIC: polymorphism information content.

**Table 2:** PCR primers and PCR-SSCP conditions used for ovine *KRTAP19-n*.

Gene ^1^	Primer Sequence (5′-3′)	Expected Size	Annealing Temperature	SSCP Condition
*19-1*	CTCCCATAAATGCACACTTTG	484 bp	58 °C	22 °C, 360 V
	TCTTAAGGTTTACATGTATACAG			
*19-2*	TTCCCACGTGTTCTGACAAG	366 bp	60 °C	22 °C, 320 V
	CATTCTTGGTAGCAGATGTTG			
*19-4*	AAGGAATGACCACATGTTTTC	416 bp	60 °C	17.5 °C, 360 V
	CAGTTCTTCAACCGAATAATG			
*19-6*	GCCTCCCGCAAATGTACAG	462 bp	58 °C	22 °C, 320 V
	AGAGTAATCTTAATTCATTGATTG			

^1^ The gene names are shown in a shortened form (i.e., *KRTAP19-1* is represented as *19-1*).

## Data Availability

The raw data will be made available by the authors on request.
